# Luminal DMSO: Effects on Detrusor and Urothelial/Lamina Propria Function

**DOI:** 10.1155/2014/347616

**Published:** 2014-05-18

**Authors:** Katrina J. Smith, Russ Chess-Williams, Catherine McDermott

**Affiliations:** Faculty of Health Sciences & Medicine, Bond University, Gold Coast, QLD 4229, Australia

## Abstract

DMSO is used as a treatment for interstitial cystitis and this study examined the effects of luminal DMSO treatment on bladder function and histology. Porcine bladder was incubated without (controls) or with DMSO (50%) applied to the luminal surface and the release of ATP, acetylcholine, and LDH assessed during incubation and in tissues strips after DMSO incubation. Luminally applied DMSO caused ATP, Ach, and LDH release from the urothelial surface during treatment, with loss of urothelial layers also evident histologically. In strips of urothelium/lamina propria from DMSO pretreated bladders the release of both ATP and Ach was depressed, while contractile responses to carbachol were enhanced. Detrusor muscle contractile responses to carbachol were not affected by DMSO pretreatment, but neurogenic responses to electrical field stimulation were enhanced. The presence of an intact urothelium/lamina propria inhibited detrusor contraction to carbachol by 53% and this inhibition was significantly reduced in DMSO pretreated tissues. Detection of LDH in the treatment medium suggests that DMSO permeabilised urothelial membranes causing leakage of cytosolic contents including ATP and Ach rather than enhancing release of these mediators. The increase in contractile response and high levels of ATP are consistent with initial flare up in IC/PBS symptoms after DMSO treatment.

## 1. Introduction

Interstitial cystitis or painful bladder syndrome (IC/PBS) is a chronic inflammatory bladder disease characterised by bladder or pelvic pain and irritative voiding symptoms in the absence of infection or other pathology [1]. However symptoms are variable and often include urinary urgency and frequency and painful urination [2]. A range of oral and intravesical therapies are available for IC/PBS; however, reliable evidence from well-designed clinical studies is generally lacking [3, 4].

Dimethyl sulfoxide (DMSO) has been used since the 1960s to treat the symptoms of IC/PBS [5]. Despite limited clinical trial data, Rimso-50 which is a 50% (v/v) solution of DMSO is used to relieve pain and reduce the inflammation and voiding symptoms observed in patients with this condition [3, 4]. The exact mechanism of action of DMSO is not fully understood; however, it is reported to cause mucosal damage and have analgesic, anti-inflammatory, bacteriostatic, and muscle relaxant properties [3, 6, 7]. Borzelleca et al. reported that 50% DMSO causes desquamation of the urothelium without altering the lamina propria [8]. Recently, studies have identified that DMSO directly affects cellular phospholipid membranes, with DMSO molecules found to occupy positions just below the lipid head groups acting as a spacer increasing average lateral distance favouring the entrance of water into the cell. Higher concentrations of DMSO directly increase lateral expansion of the cellular bilayer and have been known to cause destruction of cellular lipid bilayers [9, 10]. Patients commencing treatment with DMSO often experience an initial flare up of symptoms, which usually subsides after 2 weeks [3].

DMSO is highly permeable and will come into contact with the urothelium but possibly also the underlying lamina propria, detrusor smooth muscle, and the nerves innervating the bladder wall. The urothelium forms a barrier, protecting underlying nerves and muscle from contents of the urine; however, the urothelium and lamina propria also play important roles in bladder sensation, with the urothelium releasing a number of mediators including ATP, acetylcholine (Ach), prostaglandin E2 (PGE2), nitric oxide (NO) [11], and an unidentified diffusible substance known as urothelial-derived inhibitory factor (UDIF) [12]. These mediators are involved in modulating sensory nerve activity and also detrusor function. DMSO has been reported to depress nitric oxide release from efferent nerves [13].

All 5 muscarinic subtypes are expressed in the human urothelium and stimulation of these receptors in the urothelium releases ATP, NO, and UDIF [14, 15]. Bladder stretch during filling triggers ATP release from the urothelium and also subepithelial myofibroblasts, and this ATP is believed to be the source of primary excitation of the bladder afferents by acting on the P2X receptors [16–18]. The urothelial P2X_2/3_ receptors have been implicated in the sensory role involved with micturition and also nociception in pathological states [19, 20]. Myelinated A*δ* afferent fibres are believed to be involved in the nonpainful micturition reflex, whereas high threshold unmyelinated C afferent fibres are activated in painful, pathological conditions.

Intravesical dimethylsulfoxide (DMSO) is used for the treatment of interstitial cystitis/painful bladder syndrome (IC/PBS) although patients initially experience a flare up of symptoms on commencement of treatment. Little is known regarding the effects of treatment on bladder function or the cause of the initial worsening of symptoms. As the urothelium comes into contact with the highest concentrations of DMSO during intravesical administration, changes in urothelial function may be involved in the drugs therapeutic actions but also initial worsening of symptoms. The aim of this study was therefore to investigate possible changes in urothelial/lamina propria and detrusor function using an* in vitro* model to simulate intravesical DMSO treatment.

## 2. Materials and Methods

### 2.1. Drugs, Chemicals, and Reagents

Carbachol (carbamylcholine chloride), adenosine triphosphate (ATP), and dimethyl sulfoxide (DMSO) were obtained from Sigma-Aldrich (Castle Hill, New South Wales, Australia). Solutions were prepared in deionised water and further diluted in Krebs-bicarbonate solution.

### 2.2. Luminal Treatment of Porcine Bladder with DMSO

**Figure 1:**
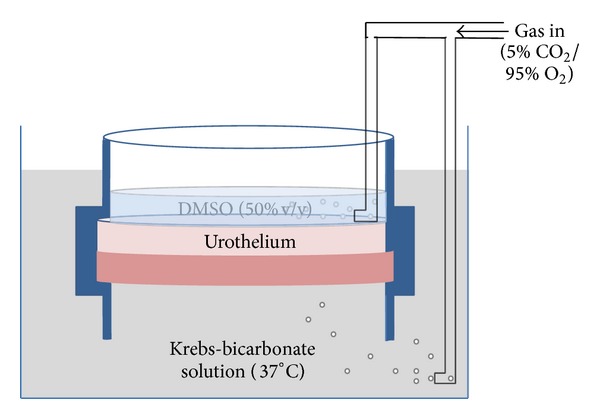
Schematic figure of the incubation chamber. Full thickness sheets of bladder dome were sandwiched between two separated bathing solutions, with each containing gassed (5% CO_2_/95% O_2_) Krebs-bicarbonate (serosal) or DMSO (urothelial) solution at 37°C. Tissues were incubated with DMSO (50% v/v) applied to the luminal side only for 15 min before isolation of the various tissues for pharmacological analysis.

Fresh bladders from Large White-Landrace pigs (6 months old, 80 kg) were obtained from a local abattoir and immediately immersed in cold Krebs-bicarbonate solution (composition in mM: NaCl 118, NaHCO_3_ 24.9, CaCl_2_ 1.9, MgSO_4_ 1.15, KCl 4.7, KH_2_PO_4_ 1.15, and D-glucose 11.7). The bladders were opened longitudinally and sheets of full thickness anterior wall from the dome region were set up in a bath where separated gassed (5% CO_2_/95% O_2_) solutions bathed the luminal and serosal surfaces (see Figure 1), allowing dimethyl sulfoxide (DMSO) to be administered to the luminal surface only, with Krebs-bicarbonate solution bathing the serosal surface. The tissues were incubated at 37°C for 15 min with a therapeutic concentration (50% v/v) of DMSO applied to the luminal surface. Control bladders were incubated for 15 min without the addition of DMSO. Incubation media were collected immediately after the 15-minute incubation to measure release of ATP, Ach, and lactate dehydrogenase (LDH) from the luminal surfaces during the treatment. After the incubation (control or DMSO), tissue strips were then isolated and set up under 1 g resting tension in organ baths containing Krebs-bicarbonate solution at 37°C to allow for the examination of tissue responses. Four sets of tissues were examined:

- full thickness bladder wall with an intact urothelium and lamina propria;
- denuded detrusor strips with the urothelium and lamina propria removed;
- strips of urothelium and lamina propria for recording of tissue contraction;
- strips of urothelium and lamina propria for the measurement of stretch-induced ATP and acetylcholine release.

### 2.3. Functional Organ Bath Studies

To assess the effects of DMSO on tissue responsiveness, contractile response to ATP (1 mM) and cumulative concentration-response curves to the muscarinic receptor agonist carbachol (1 nM–10 *μ*M) were obtained on tissues (i), (ii), and (iii). Isometric contractions of isolated tissue strips were recorded using a Powerlab data acquisition system (ADInstruments).

To investigate the effects of DMSO on nerve mediated responses, detrusor strips denuded of urothelium and lamina propria (tissues (ii)) were set up under 1 g resting tension in organ baths and electrically field stimulated via silver electrodes placed either side of the tissue. Tissues were stimulated at 1, 5, 10, and 20 Hz using 5 s trains of pulses (20 v, 0.5 ms pulse-width) delivered every 100 s. Contractile responses and the release of mediators for tissues from DMSO pretreatment bladders were compared with those of tissues from control incubated bladders.

Tissues (iv) were used to examine the effects of DMSO on basal and stretch-induced release of mediators from the urothelium/lamina propria. These tissues were washed and 2 min later a sample of the bathing medium was collected and frozen for later assay of mediators (basal release). The tissues were then stretched, increasing length by 50%, and the bathing medium again was collected and frozen for assay of mediators (stimulated release).

### 2.4. Measurement of ATP, Ach, and LDH

ATP was measured using a luciferase-luciferin assay kit (Molecular Probes) according to the manufacturer's instructions. Luminescence was measured using a Modulus microplate reader (Promega). Acetylcholine was measured using a fluorescence-based Amplex Red Acetylcholine Assay kit (Molecular Probes) according to the manufacturer's protocol. Fluorescence was measured on a Modulus Microplate reader (Ex. 540/Em. 590 nm). Leakage of LDH into the incubation media was measured using LDH Cytotoxicity assay (Cayman Chemicals). Absorbance was measured on a Modulus Microplate reader (490 nm).

### 2.5. Bladder Histology

Sections of control and DMSO pretreated intact bladder dome were fixed (4% neutral buffered formalin), processed, and embedded in paraffin. Tissues were sectioned at 6 *μ*m and placed on uncharged slides. Sections were stained using haematoxylin and eosin to assess urothelial integrity and examined using an Olympus CX31 microscope (Olympus Australia Pty. Ltd.) equipped with an Infinity 2 camera and Infinity Capture software. Image J software was used to measure relative urothelial thickness in control and DMSO pretreated tissues.

### 2.6. Data Analysis and Statistical Procedures

Mean (± SEM) increases in tension induced by carbachol or electrical field stimulation were calculated. For responses to carbachol, individual −Log EC50 (pEC50) values were determined from the concentration-response curves by the use of GraphPad Prism 5 software (SanDiego, CA) and mean (± SEM) pEC50 values and maximum responses were calculated. Similarly, for the mediator release study, mean (± SEM) concentrations were determined before and after stretch and data for DMSO and control pretreated bladders were compared. Data were analysed using a paired Student's* t*-test or one-way ANOVA with Dunnett's multiple comparisons test, using Graphpad InStat3 software (SanDiego, CA). Significance levels were defined as *P* < 0.05. *n* values represent the number of separate pig bladders examined.

## 3. Results

### 3.1. Mediator Release during Incubations

**Figure 2:**
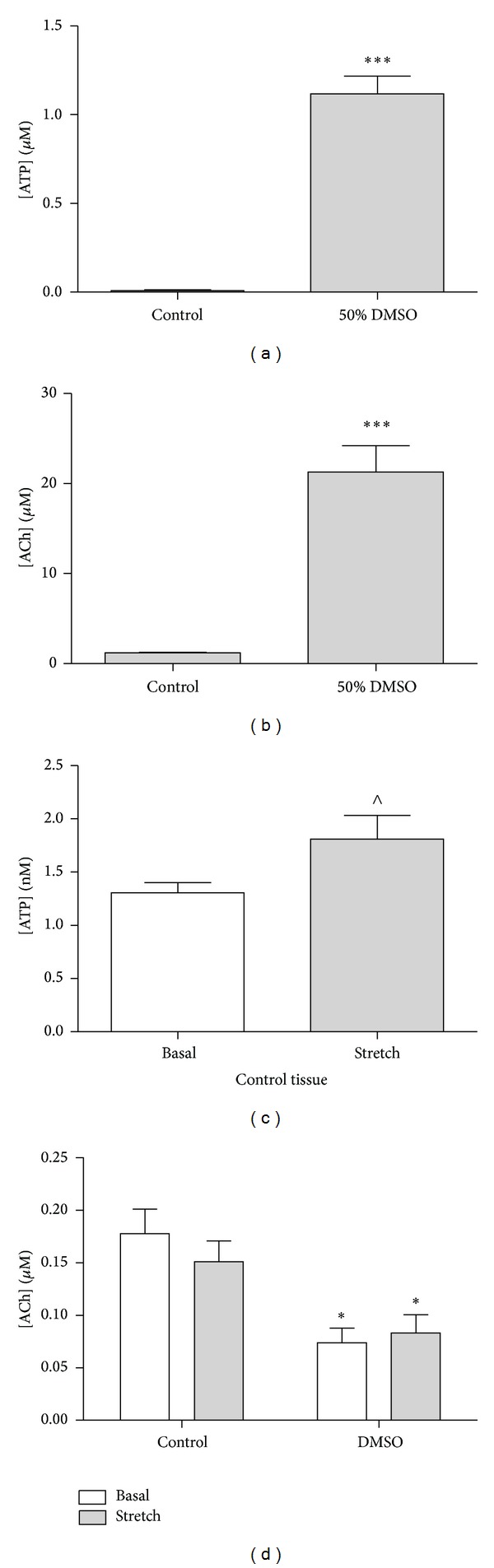
Effect of DMSO (50% v/v) on (a) ATP and (b) Ach release into incubation medium during bladder pretreatment and also on subsequent basal and stretch-induced release of (c) ATP and (d) Ach from strips of urothelium/lamina propria. Data represents mean ± SEM. (***P < 0.001, DMSO pretreated versus control incubated bladders. ^∧^ *P* < 0.05, control basal versus control stretch. *P < 0.05, DMSO pretreated versus control incubated tissues).

At the end of the pretreatment period the incubation medium was collected and assayed for Ach (*n* = 5) and ATP (*n* = 4). Concentrations of Ach were significantly greater than those of ATP during both control (1.22 ± 0.05 *μ*M versus 0.010 ± 0.003 *μ*M, *P* < 0.001) and DMSO incubations (21.3 ± 2.94 *μ*M versus 1.12 ± 0.09 µM, *P* < 0.001). The presence of DMSO during the incubation produced a significant increase in the levels of both mediators (Figures 2(a) and 2(b)), Ach levels rising 17-fold (*P* < 0.001), and ATP levels rising by >100-fold (*P* < 0.001). LDH (7 mU/mL) was also detected in luminal incubation medium from DMSO treated bladders, but none was detected in matched controls.

### 3.2. Mediator Release from the Urothelium/Lamina Propria after Incubation

Isolated strips of urothelium/lamina propria prepared from control incubated bladders released both ATP (*n* = 4) and Ach (*n* = 8) under basal and stretch conditions. There was a significant increase in ATP release in response to stretch (Figure 2(c)) in control tissues; however, basal and stretch-induced ATP release from DMSO treated tissues were not detected. Ach was released from control urothelium/lamina propria under basal conditions with no significant increase in response to stretch. Ach release from DMSO treated tissue was significantly reduced (Figure 2(d)).

### 3.3. Contractile Responses following Incubation with DMSO

**Figure 3:**
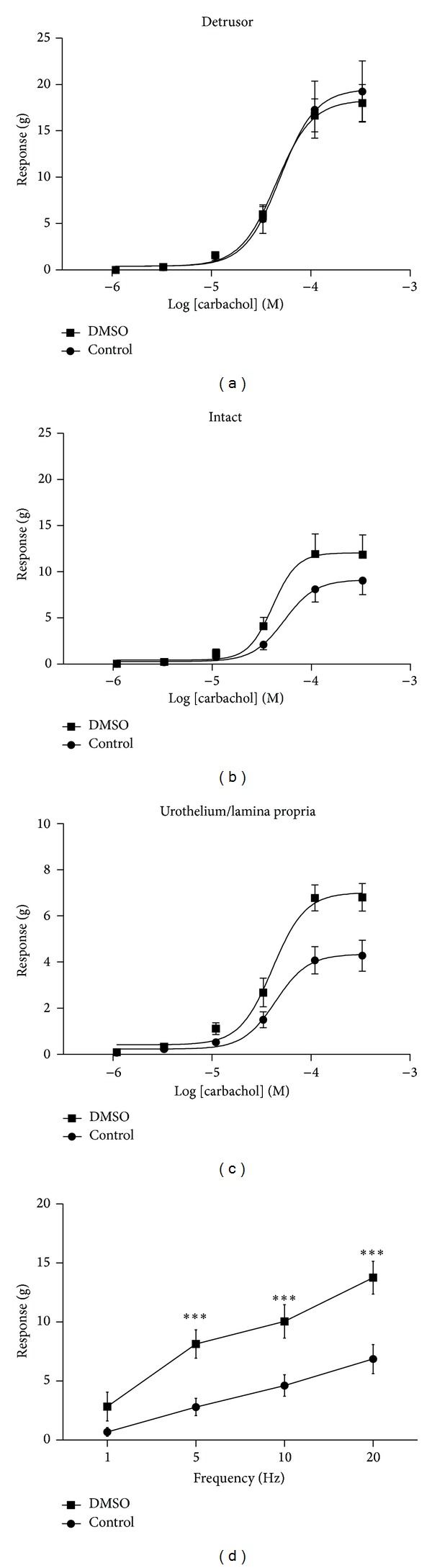
Cumulative carbachol concentration-response curves (*n* ≥ 4) for DMSO (50% v/v) preincubated and control preincubated strips of (a) denuded detrusor, (b) intact tissue, and (c) urothelium/lamina propria. Detrusor strip responses to electrical field stimulation (d) are also shown (*n* = 5). Data represents mean ± SEM. ***P < 0.001 compared to responses of control incubated tissues.

Luminal pretreatment of bladders with DMSO (50%) did not significantly affect detrusor, urothelial, or intact tissue response to KCl or ATP (*n* = 5, data not shown). Similarly, DMSO pretreatment did not affect subsequent responses of isolated detrusor smooth muscle strips to the muscarinic receptor agonist carbachol (Figure 3(a), *n* = 4), with both pEC50 values and maximum responses to carbachol being similar in muscle strips from DMSO and control pretreated bladders (Table 1). However responses of urothelium/lamina propria strips and the responses of intact bladder strips (detrusor plus urothelium/lamina propria, *n* = 5) to carbachol were enhanced after pretreatment with DMSO (Figures 3(b) and 3(c), Table 1).

The presence of the urothelium/lamina propria in the intact tissues significantly inhibited contractions of bladder strips (Figure 3, Table 1). This inhibition was significantly (*P* < 0.05) greater in control tissues (53 ± 7.8%) than in tissues from DMSO pretreated bladders (33 ± 4.1%).

Detrusor responses to electrical field stimulation (EFS) were frequency dependent and contractions were increased in tissues from DMSO pretreated bladders. Contractions to EFS were greater at all stimulation frequencies examined, with the differences being statistically significant for the responses at 5 Hz, 10 Hz, and 20 Hz (Figure 3(d), *n* = 5). In the presence of atropine (1 *μ*M), detrusor contractions to EFS were depressed by 68 ± 10% at 20 Hz in control tissues (*P* < 0.001,   *n* = 5). The inhibition of responses to EFS by this muscarinic antagonist was similar at all frequencies examined and was not altered significantly by DMSO pretreatment (75 ± 6.1% inhibition at 20 Hz, *n* = 5).

### 3.4. Bladder Histology

**Figure 4:**
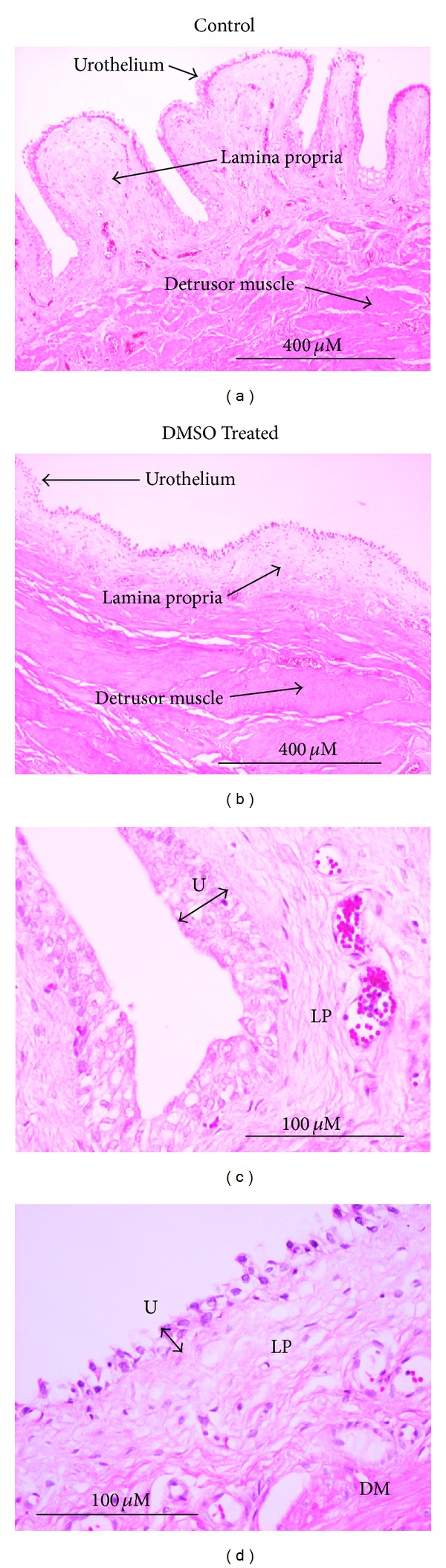
Hematoxylin and eosin (H and E) staining of control (a) and (c) and DMSO pretreated (b) and (d) bladders. H and E staining at (a) and (b) 10x and (c) and (d) 40x.

Representative H and E stained sections of control and DMSO pretreated bladders (detrusor + urothelium/lamina propria) are shown (Figure 4). Typical histological features were clearly identifiable in sections of control incubated bladder, with the urothelium and lamina propria thrown into folds and overlying a deeper smooth muscle layer. However in DMSO pretreated tissues, damage to the luminal layers was evident. Urothelial thickness was significantly reduced from 31.1 ± 1.48 *μ*m in control tissues to 11.3 ± 0.45 *μ*m in DMSO treated tissues (*n* = 15,   *P* < 0.001). In addition, the folding of the urothelium/lamina propria observed in control bladders was absent from DMSO pretreated bladders.

## 4. Discussion

Although the exact mechanism by which DMSO relieves symptoms associated with IC/PBS is unclear, when applied to the human skin, it penetrates rapidly and produces pharmacological effects such as anti-inflammation, analgesia, and bacteriostasis [21]. For IC/PBS, DMSO is administered intravesically and, due to the highly absorptive nature of DMSO, it is likely that not only the urothelium but also the underlying lamina propria, detrusor smooth muscle, and nerves innervating the bladder wall will be affected by DMSO. However, it is the urothelial layer that comes into direct contact with DMSO and is subject to the highest concentrations.

Treatment with DMSO is usually biweekly for 3 months and this has been found to be effective for approximately 16 to 72 months [22]. Previous studies have reported urothelial desquamation, mucosal damage, and interference of cellular phospholipid membranes to be associated with the application of DMSO [3, 6–10]. Chemical injury and subsequent loss of urothelial layers exposed to DMSO over a 3-month period may explain some of its effectiveness in treating IC/PBS as it has been reported that the removal of a diseased urothelium by laser treatment leads to nonrecurrence of pain for 6–12 months [23]. In the present study, loss of urothelial layers and mucosal folding were also detected following DMSO treatment. In addition, the detection of LDH in the luminal treatment effluent suggests that DMSO permeabilised the urothelial membranes causing leakage of cytosolic contents. The high levels of ATP and Ach detected in the treatment effluent may therefore reflect leakage of these mediators from the urothelium due to physical damage rather than enhanced levels of release.

High levels of ATP and Ach were observed during luminal treatment with DMSO, and urothelial mediator release after treatment was also investigated. Following exposure to DMSO neither subsequent basal release nor stretch-induced release of ATP could be detected. Ach release was also significantly reduced following DMSO treatment. This is likely due to depletion of ATP and Ach stores during DMSO treatment and loss of urothelial cells from the mucosal surface. The inhibition of ATP release in response to stretch from urothelium treated with DMSO is consistent with previous reports which have identified a significant decrease in ATP release from the urothelium treated with DMSO in response to stretch [24].

Augmented release of urothelial ATP and changes to urothelial purinergic P2X and P2Y receptor profiles are common features of patients suffering from IC/PBS [14]. It is well established that ATP, released in response to stretch, acts on afferent nerve P2X_2/3_ receptors, playing a sensory role in the micturition cycle and also nociception in pathological states [20]. Therefore, lower levels of urothelial ATP release after treatment with DMSO may be beneficial in correcting the augmented ATP release and the enhanced afferent nerve activity observed in IC/PBS.

It has been reported that in the porcine and human bladder the urothelium/lamina propria releases a factor that inhibits detrusor contraction [12, 25]. This inhibitory effect of the urothelium was evident in tissues from both control and DMSO pretreated bladders, but the inhibitory effect was significantly reduced following DMSO pretreatment. The consequences of this change are unknown, but a similar reduction in inhibitory mechanisms has been observed in the human neurogenic overactive bladder [26]. Thus the observed reduction in urothelial inhibition of the detrusor may contribute to the bladder overactivity observed after DMSO treatment. Surprisingly urothelial contractile responses to carbachol were enhanced following DMSO pretreatment. It has been suggested that this activity is responsible for correct folding of the urothelium on bladder emptying [27] or contractile activity of this layer may drive detrusor contraction [28] but the clinical relevance of this urothelial activity is currently unknown.

Detrusor responses to carbachol were not affected by DMSO pretreatment suggesting that only lower concentrations of DMSO permeate to these deeper tissues. However detrusor responses to EFS were altered by pretreatment which indicates that the parasympathetic nerves in this tissue are more sensitive to the actions of DMSO than the detrusor muscle itself. Unexpectedly, responses to EFS were enhanced by DMSO pretreatment. Since detrusor responses to exogenous carbachol were unchanged, this suggests that DMSO increases neurotransmitter release to EFS. The enhanced responses may be the result of DMSO causing damage to the nerve terminals which may ease when depletion of these stores has been completed. This is consistent with previous research which also noted acute reflex firing of pelvic nerve efferent axons in response to DMSO [13]. Atropine inhibited responses to EFS similarly in control and DMSO pretreated tissues, suggesting that the cholinergic contribution to neurotransmission did not change following treatment.

The acute administration of DMSO has been found to cause irritation [29] and the associated pain is reportedly caused by mast cell degranulation, in response to chemical injury which eases on depletion [30]. The increase in contractile responses and high levels of ATP are consistent with initial flare up in IC/PBS symptoms after DMSO treatment.

In conclusion, this study demonstrates a physical and functional disruption of the bladder urothelium/lamina propria following luminal exposure to DMSO. During treatment there was a large release of mediators from the urothelium/lamina propria which are known to trigger micturition and initiate sensations of pain. These sensations would be greatly enhanced by the increases in muscarinic and purinergic receptors previously reported for patients with IC/PBS. The detection of LDH in the treatment medium suggests that release was due to permeabilisation of the urothelial membranes rather than stimulated physiological release. These effects on mediators and also the reduced inhibitory role of the urothelium/lamina propria on detrusor contraction following DMSO pretreatment may contribute to the initial flare up in symptoms experienced by most patients following intravesical DMSO treatment. Thus antagonists of sensory nerve purinergic receptors currently in development may potentially offer relief during this initial period after DMSO treatment.

## Figures and Tables

**Table 1:** Mean (±SEM) maximal responses (g) and pEC50 values for carbachol on tissue strips prepared from DMSO or control pretreated bladders.

Carbachol	Urothelium/lamina propria		Detrusor smooth muscle		Intact bladder strips	
	Control	DMSO	Control	DMSO	Control	DMSO
Maximum response (g)	4.2 ± 0.28	6.6 ± 0.35***	17.8 ± 1.4	17.1 ± 0.7	8.3 ± 0.63	11.5 ± 0.94*
pEC_50_	4.14 ± 0.09	4.4 ± 0.08	4.37 ± 0.12	4.39 ± 0.07	4.35 ± 0.14	4.45 ± 0.52

*P < 0.05 and ***P < 0.001 when comparing control versus DMSO (50% v/v).

## Abbreviations

Ach: Acetylcholine
ATP: Adenosine triphosphate
DMSO: Dimethyl sulfoxide
EFS: Electric field stimulation
IC/PBS: Interstitial cystitis/painful bladder syndrome
LDH: Lactate dehydrogenase
PGE2: Prostaglandin E2
UDIF: Urothelial-derived inhibitory factor.
